# Gut microbiota depletion by antibiotics ameliorates somatic neuropathic pain induced by nerve injury, chemotherapy, and diabetes in mice

**DOI:** 10.1186/s12974-022-02523-w

**Published:** 2022-06-28

**Authors:** Pingchuan Ma, Rufan Mo, Huabao Liao, Chengjie Qiu, Genhao Wu, Caixia Yang, Yunxiao Zhang, Yiran Zhao, Xue-Jun Song

**Affiliations:** 1 Department of Medical Neurocience and SUSTech Center for Pain Medicine, School of Medicine, Southern University of Science and Technology, 1088 Xueyuan Ave, Nanshan District, Shenzhen, 518055 Guangdong China; 2 Key Laboratory of Carcinogenesis and Translational Research (Ministry of Education of China), Peking University Cancer Hospital and Institute, Beijing, 100142 China

**Keywords:** Gut microbiota, Neuropathic pain, Chemotherapy, Diabetes

## Abstract

**Background:**

Gut microbiota has been found involved in neuronal functions and neurological disorders. Whether and how gut microbiota impacts chronic somatic pain disorders remain elusive.

**Methods:**

Neuropathic pain was produced by different forms of injury or diseases, the chronic constriction injury (CCI) of the sciatic nerves, oxaliplatin (OXA) chemotherapy, and streptozocin (STZ)-induced diabetes in mice. Continuous feeding of antibiotics (ABX) cocktail was used to cause major depletion of the gut microbiota. Fecal microbiota, biochemical changes in the spinal cord and dorsal root ganglion (DRG), and the behaviorally expressed painful syndromes were assessed.

**Results:**

Under condition of gut microbiota depletion, CCI, OXA, or STZ treatment-induced thermal hyperalgesia or mechanical allodynia were prevented or completely suppressed. Gut microbiota depletion also prevented CCI or STZ treatment-induced glial cell activation in the spinal cord and inhibited cytokine production in DRG in OXA model. Interestingly, STZ treatment failed to induce the diabetic high blood glucose and painful hypersensitivity in animals with the gut microbiota depletion. ABX feeding starting simultaneously with CCI, OXA, or STZ treatment resulted in instant analgesia in all the animals. ABX feeding starting after establishment of the neuropathic pain in CCI- and STZ-, but not OXA-treated animals produced significant alleviation of the thermal hyeralgesia or mechanical allodynia. Transplantation of fecal bacteria from SPF mice to ABX-treated mice partially restored the gut microbiota and fully rescued the behaviorally expressed neuropathic pain, of which, Akkermansia, Bacteroides, and Desulfovibrionaceae phylus may play a key role.

**Conclusion:**

This study demonstrates distinct roles of gut microbiota in the pathogenesis of chronic painful conditions with nerve injury, chemotherapy and diabetic neuropathy and supports the clinical significance of fecal bacteria transplantation.

## Background

Gut microbiota, which represents the massive commensal microbes colonizing the gastrointestinal (GI) tract, has been demonstrated to play important roles in many physiological and pathological processes of our bodies [1]. Resident in the gut, gut microbiota is able to regulate local mucosal immune functions of the gut [2], GI tract motility [3], and some local neuronal processes like visceral pain sensations [4]. What’s more, evidence starts to uncover the capacity of gut microbiota in regulating some functions in distal organs, among which the nervous system functions are of great interest [5]. The so-called “microbiota–gut–brain” axis, which refers to the interactions among microbiota, gut and the brain through circulation of microbial metabolites or through vagus nerve transmission, are found to regulate the neurodevelopment [6, 7], neurotransmitter release [8], and glial functions in the brain [9]. These interactions also modulate animal behaviors as well as the progression of diseases like depression [10], Alzheimer’s disease [11], and autism spectrum disorders [7]. However, it remains largely unknown how gut microbiota interacts with the peripheral nervous system and chronic pain, especially chronic somatic pain disorders, which have been major clinical problems for a long time.

Previous studies have shown that gut microbiota participates in the development of inflammation pain [12] and chemotherapy-induced neuropathic pain [13]. Gut microbiota regulates the cytokine productions in the spinal cord in inflammation pain and impacts the dorsal root ganglion (DRG) neuron functions through releasing lipopolysaccharides (LPS) into the circulation in chemotherapy-induced neuropathic pain. Different forms of chronic pain share some common symptoms and pathogenesis but also have distinct features. Different pain modalities such as thermal pain and mechanical pain has distinct molecular and neuronal transduction pathways [14]. Despite of the few findings regarding gut microbiota’s role in pain, there’s a lack of systematic studies comparing effects of gut microbiota on different forms of chronic pain conditions, as well as on different pain modalities. In this study we investigated roles of gut microbiota in neuropathic pain induced by nerve injury, chemotherapy, and diabetes, as well as in different pain modalities including thermal and mechanical pain. We found that the production and persistence of neuropathic pain induced by nerve injury, chemotherapy, or diabetes, respectively, can be prevented or greatly suppressed by gut microbiota depletion and rescued following gut microbiota restoration by transplanting fresh fecal bacteria. This study indicates an important and distinct role of gut microbiota in different forms of chronic pain conditions and may suggest gut microbiota as a therapeutic or regulatory target for treating neuropathic pain.

## Methods

### Animals, drugs, and drug administration

We purchased conventional SPF C56BL/7 mice (6 weeks) from the Experimental Animal Center, Southern University of Science and Technology (SUSTech). All protocols were approved by the Animal Care and Use Committee of SUSTech. All surgeries were done under anesthesia with Avertin (350 mg/kg, i.p.). For gut microbiota eradication, SPF mice were provided, ad libitum, with drinking water containing antibiotic cocktail with 0.5 g/L ampicillin 0.5 g/L neomycin, 0.5 g/L metronidazole, and 0.25 g/L vancomycin [13] (all antibiotics were from Sigma-Aldrich, St. Louis, MO, USA). Antibiotics water was maintained during the entire experimental period for mice except for the restoring period of gut microbiota. Antibiotics were renewed every two days. For the fecal transplantation experiment, freshly collected feces from SPF mice were diluted 1:10 with PBS and oral gavaged to ABX-treated mice with 0.3 ml per mouse daily for 3 days, at the same time, ABX water was replaced with regular water. To mimic chemotherapy-induced pain, oxaliplatin (OXA, SAGENT pharmaceuticals, Schaumburg, IL) was intraperitoneally injected (i.p.) at 3 mg/kg for five consecutive days. To produce diabetic neuropathic pain, after fasting for 12 h, the mice received streptozocin (STZ, from Sigma-Aldrich) with i.p. 40 mg/kg per day for 5 consecutive days. Blood glucose was determined by blood glucose meter (Sannuo, Changsha, China).

### Animal models of neuropathic pain induced by nerve injury, chemotherapy and diabetes

Chronic constriction injury of the sciatic nerve (CCI) has been a widely used mode of neuropathic pain after peripheral nerve injury in rodents [15]. In brief, the left common sciatic nerve of each mouse employed was exposed at the mid-thigh level. Proximal to the sciatic nerve’s trifurcation was freed of adhering tissue and three silk ligatures (surgical silk 1.5) were tied loosely around with approximately 1 mm between ligatures. Animals in the sham group received surgery identical to that described in CCI treatment but without nerve injury. Chemotherapy-induced neuropathic pain was induced by OXA treatment [13]. Diabetes and diabetic neuropathic pain were induced by STZ treatment [16].

### Assessment of mechanical allodynia and thermal hyperalgesia

The mechanical pain thresholds were measured at different time points before and after various treatments in the animals. Mechanical allodynia was determined by measuring the threshold of foot withdrawal in response to mechanical stimulus of each hind-paw using a sharp, cylindrical probe with a uniform tip diameter of 0.2 mm from an Electro von Frey [17] (ALMEMO 2390-5 Anesthesiometer; IITC Life Science, Woodland Hills, CA). The probe was applied to 6 designated loci distributed over the plantar surface of the foot. The minimal force (in grams) that induced paw withdrawal was read off of the display. Threshold of mechanical withdrawal in each animal was calculated by averaging the 6 readings, and the force was converted into milli-Newtons (mN). The results represent the mean values of ipsilateral feet.

Thermal hyperalgesia was assessed by measuring foot withdrawal latency to heat stimulation[15]. An analgesia meter (IITC Model 336 Analgesia Meter, Series 8; IITC Life Science) was used to provide a heat source. Each animal was placed in a box containing a smooth, temperature-controlled glass floor. The heat source was focused on a portion of the hind-paw, which was flush against the glass, and a radiant thermal stimulus was delivered to that site. The stimulus shut off when the hind-paw moved (or after 30 s to prevent tissue damage). The intensity of the heat stimulus was maintained constant throughout all experiments. The intensity of the heat stimulus was maintained constant throughout all experiments. The elicited paw movement occurred at latency between 5 and 10 s in control animals. Thermal stimuli were delivered 3 times to each hind-paw at 5–6 min intervals. For the results expressing mechanical allodynia or thermal hyperalgesia, the values are mean of ipsilateral feet. Experimenters who performed both mechanical and thermal behavioral tests were always blinded to the treatment conditions.

### Measurement of capsaicin-induced licking behavior [18]

Mice were habituated for 30 min per day consecutively for 3 days before testing. Left hind-paw of mice were intraplantar injected with 3 μg/10 μL capsaicin (Sigma-Aldrich). Mice were video-recorded for 10 min to determine licking durations.

### CFU counting of mice feces

The feces from mice were collected in disinfected tubes with 1 mL sterile PBS. After homogenating the mixture was diluted by 1000 times and 40 μL was plated on TSA media plate. The plates were placed in 37 °C overnight and the CFU on each plate was counted.

### DNA sequencing and analysis

Library preparations for DNA sequencing and Illumina MiSeq sequencing were conducted at GENEWIZ, Inc. (Suzhou, China). Fecal DNA was extracted with DNA Stool Kit (QIAGEN, Hilden, Germany) according to the manufacturer’s instruction. DNA samples were quantified by using Qubit 2.0. Fluorometer (Invitrogen, Carlsbad, CA, USA). 50–100 ng DNA was used to generate amplicons using the GENEWIZ prepared kit. V7 and V8 hypervariable regions of prokaryotic 16S rDNA were selected for generating amplicons and following the taxonomy analysis. The v7 and v8 regions were amplified using forward primers containing the sequence “CGWTAACGAACGAG” and reverse primers containing the sequence “AICCATTCAATCGG”. At the same time, indexed adapters were added to the ends of the 18S rDNA amplicons to prepare for downstream NGS sequencing on Illumina Miseq. DNA libraries were validated by Agilent 2100 Bioanalyzer (Agilent Technologies, Palo Alto, CA, USA) and quantified by Qubit 2.0 Fluorometer. DNA libraries were multiplexed and loaded on an Illumina MiSeq instrument according to the manufacturer’s instructions (Illumina, San Diego, CA, USA). Sequencing was performed using a PE300/250 paired-end configuration; image analysis and base calling were conducted by the MiSeq Control Software (MCS) embedded in the MiSeq instrument. OTUs were clustered by using VSEARCH (1.9.6), and then OTU rank curse was generated by RDP classifier (Ribosomal Database Program). The Shannon index could be calculated based on the analysis of OTU.

### Protein determination

To qualify temporal changes in protein levels, Western blotting analysis was used. The spinal cord and DRGs at L4–L6) were quickly removed from deeply anesthetized mice and stored at − 80 °C. Sequential precipitation procedures were used on the tissue samples and were lysed in ice-cold (4 °C) RIPA lysis buffer containing a mixture of protease inhibitor, phosphatase inhibitors, and phenylmethylsulfonyl fluoride (Sigma-Aldrich). The total protein was separated by SDS-PAGE and transferred to PVDF membrane (both from Bio-Rad Laboratories, Hercules, CA, USA). The following primary antibodies were used: anti-Interleukin 6 (1:200; Santa Cruz, Santa Cruz, CA, USA), anti-Tumor necrosis factor alpha (1:200; Santa Cruz), anti-GAPDH (1:1000; Sangon, Shanghai, China). The membranes were then developed by enhanced chemiluminescence reagents with horseradish peroxidase-conjugated secondary antibodies (R&D Systems, Minneapolis, MN, USA). Images were acquired with Tanon chemiluminescence instrument (Shanghai, China). Data were analyzed with the ImageJ. Absolute gray level of each plot is quantified with background subtraction and then normalized with the control plot (GAPDH) for comparison.

### Immunohistochemistry

Deeply anesthetized mice were perfused transcardially with 0.9% saline followed by 4% paraformaldehyde. The spinal cord and DRGs at L4–L6 segments were removed and postfixed in 4% paraformaldehyde 24–48 h. After being postfixed, the tissues were transferred into 40% sucrose (in 0.1 M PB) for 3 d for dehydration. The tissues were sectioned at 20 μm thickness for spinal cord and DRG sections. For immunofluorescence staining, free-floating sections were blocked in PBS containing 10% donkey serum with 0.3% Triton X-100 for 2 h and incubated in primary antibody at 4 °C overnight. Sections were then washed in 0.1 M PBS with 0.05% Triton X-100, pH 7.6 (3 × 5 min) followed by incubating in the secondary antibody at room temperature for 2 h and washing. Sections were mounted on slides and covered with 90% glycerin for observation under a confocal microscope (AR1; Nikon, Tokyo, Japan). The antibodies used included anti-glial fibrillary acidic protein (1:500; Millipore, Burlington, MA), anti-IBA1(1:500; FUJIFILM Cellular Dynamics, Madison, WI, USA).

### Stool genomic DNA concentration determination

To qualify stool DNA concentration of mice, Stool genomic DNA kit (EE301-01; Transgene, Lyon, France) was used. The stool was collected in disinfected box and quickly transferred to freezer to save. Briefly, 200 mg feces were homogenized in 2 mL tube with 0.25 g glass beads. After microbial cell lysis and remove of non-microbial cell, the DNA containing tube was eluted by elution buffer. Quantification of DNA concentration was measured using nanodrop.

### Measurement of 5-HT in the spinal cord and serum

The spinal cord at L4–L6 segments were collected and save at -80 °C. Sequential precipitation procedures were used on the tissue samples and were lysed in ice-cold (4 °C) RIPA lysis buffer containing a mixture of protease inhibitor, phosphatase inhibitors, and phenylmethylsulfonyl fluoride (Sigma-Aldrich). The blood was collected and centrifuged at 3000 rpm at 4 °C. After centrifuge, the supernatant was stored at − 80 °C. The enzyme-linked immunosorbent assay kits (ELISA, Jianglai, Shanghai, China) were used to determine the concentrations of 5-HT at the spinal cord and serum.

### Statistics

Prism (GraphPad, San Diego, CA, USA) was used to conduct all statistical analyses. Alterations of expression of the proteins detected and the behavioral responses to mechanical and thermal stimuli over time among groups were tested with one-way or two-way analysis of variance with repeated measures followed by multiple comparison tests. Individual Student’s *t*-tests were used to test specific hypotheses about differences between each operated or drug-treated group and its corresponding control group between the naive or sham and the treatment group. All data are presented as mean ± SEM.

## Results

### Antibiotics cocktail treatment causes major depletion of gut microbiota and does not alter cutaneous thermal and mechanical sensitivity in naïve animals

**Fig. 1:**
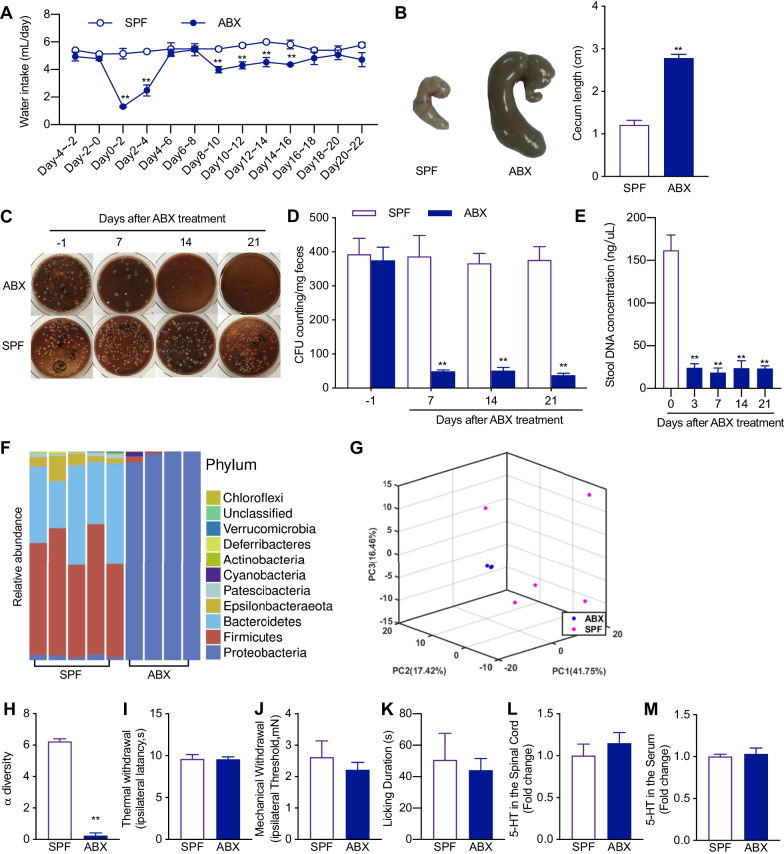
The continuous feeding of antibiotics (ABX) cocktail depletes majority of gut microbiota without affecting the behaviorally expressed thermal and mechanical sensory sensitivity in naïve mice. **A** Time course of the water intake in animals with SPF- and ABX-treatment. Two-way ANOVA with Sidak multiple comparison test **p < 0.01 (*n* = 3 in each group). **B** Representative images and statistical summary of the cecum from SPF and ABX-treated mice. The enlarged cecum and dark cecal content were shown in ABX-treated mice. Unpaired *t*-test **p < 0.01 (*n* = 6 in each group). **C**, **D** Representative images (**C**) and statistical summary (**D**) of colony-formation units (CFUs) of the fecal microbiota plated on TSA media. CFUs were reduced at day 7 and remained low at day 14 and 21 following ABX treatment. Two-way ANOVA with Sidak multiple comparison test, **p < 0.01 versus SPF (*n* = 6 in each group). **E** Effect of ABX treatment on the stool DNA concentration. One-way ANOVA with Dunnett multiple comparison test **p < 0.01 (*n* = 2–3). **F** Stacked bar-chart plot of the fecal microbiota composition of SPF and ABX-treated mice at phylum level. **G** The Principal Component Analysis of gut microbiota composition of SPF and ABX-treated mice (ABX: *n* = 4; SFP: *n* = 5). **H** 16S rRNA sequencing of fecal microbiota showing that the α-diversity of the microbiota was reduced in ABX-treated mice, compared to SPF mice. Unpaired *t*-test **p < 0.01(ABX: *n* = 4; SPF: *n* = 5). **I**–**K** ABX treatment did not alter the thermal (**I**) and mechanical sensory sensitivity (**J**) and capsaicin-induced licking (**K**) behavior. Unpaired *t*-test (*n* = 5 in each group). **L**, **M** 5-HT concentration in the spinal (**L**) and serum (**M**) of the SPF and ABX-treated mice. Unpaired *t*-test (**L** *n* = 8; **M** *n* = 5)

To explore the effects of gut microbiota on somatic pain, we first fed the mice with a combination of four different kinds of antibiotics (ABX): vancomycin (0.5 g/L), ampicillin (1 g/L), neomycin (1 g/L) and metronidazole (1 g/L), targeting different kinds of bacteria. This antibiotics cocktail was dissolved in the drinking water with free access to mice. Additional sweetener was added to prevent the aversion of mice to ABX water. The mice still showed decreased consumption of the ABX water during the first 6 days after ABX feeding, but showed no preference between normal water and ABX water after 6 days (Fig. 1A). The success of ABX feeding on the gut microbiota depletion was first confirmed by the maladaptive GI tract morphology. As previously described [19], ABX-treated mice exhibited enlarged caeca with dark-colored cecal contents as a consequence of the absence of gut microbiota (Fig. 1B). To further examine the effects of ABX feeding on gut microbiota, we collected the feces of mice and tested the fecal bacteria by plating on bacteria growth media. The colony-formation unit (CFU) counting from fecal bacteria plated on tryptic soy agar (TSA) media started to decrease at day 7 and remained significantly low at day 14 and 21 after the start of ABX feeding (Fig. 1C, D). The CFUs on TSA media only captured the change of some aerobic and facultative anaerobic bacteria of the microbiota [20]. To fully examine the change of gut microbiota, we measured the total fecal bacteria DNA concentration and performed 16S rRNA sequencing on the fecal microbiota of the mice at day 14 after the start of ABX feeding (Fig. 1E–H). The fecal bacteria DNA concentration significantly decreased at day 3 after the starting of ABX treatment and remained low until the last test at day 21 (Fig. 1E). The sequencing results showed that the composition of the microbiota was simplified, changing from a highly diverse community to a Proteobacteria phylum dominating community (Fig. 1F). The α-diversity of the microbiota was significantly decreased (Fig. 1H) after ABX feeding. The principal component analysis (PCA) showed a highly uniformized microbiota after ABX treatment and a complete division between SPF and ABX-treated microbiota (Fig. 1G). These results showed that ABX feeding caused major depletion of the gut microbiota.

We then examined the painful behaviors in ABX-treated mice. ABX-treated mice showed no difference in the cutaneous thermal and mechanical sensitivity compared with the control SPF mice (Fig. 1I, J). ABX treatment also showed no effect on subcutaneous injection of capsaicin-induced acute pain (Fig. 1K). Gut enterochromaffin cells (ECs) produce the majority of serotonin of our body [8], which is a very important neurotransmitter and neuromodulator in pain sensation [21]. Commensal bacteria in the gut are capable of promoting the biosynthesis of serotonin by ECs [8]. In consideration of these interactions, we also compared levels of serotonin in ABX-treated and SPF control mice. The results showed that gut microbiota depletion (14 days after ABX treatment) did not change serotonin concentrations in blood and the spinal cord (Fig. 1L, M).

### Gut microbiota depletion ameliorates thermal hyperalgesia and inhibits spinal glial cell activation in animals with nerve injury

**Fig. 2:**
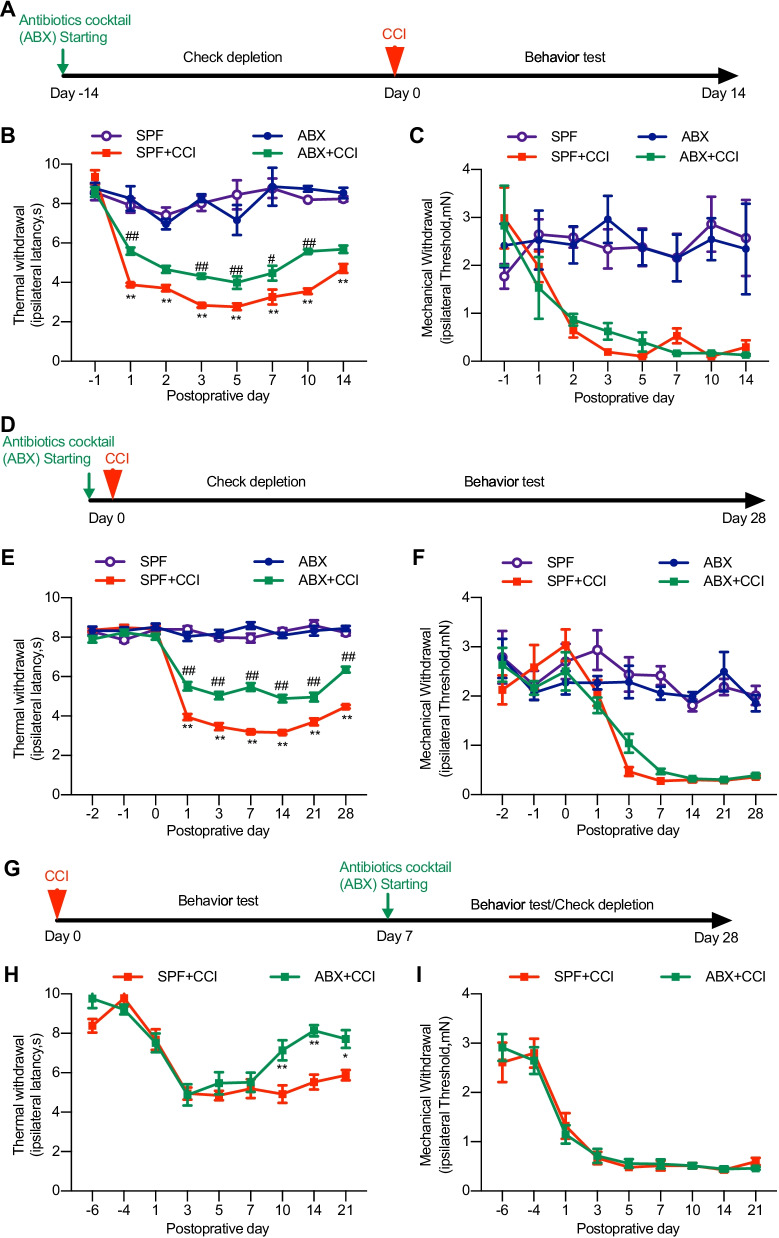
The continuous feeding of ABX alleviates CCI-induced thermal hyperalgesia but not mechanical allodynia. **A**–**C** Pre-depletion of gut microbiota, two weeks earlier than CCI treatment. **D**–**F** The continuous feeding of ABX starting simultaneously with CCI treatment. **G**–**I** The continuous feeding of ABX starting two weeks after CCI treatment when the thermal hyperalgesia and mechanical allodynia had been established. **B**, **E** Two-way ANOVA with Dunnett multiple comparison test **p < 0.01 SPF + CCI versus SPF, ^##^*p* < 0.01 SPF + CCI versus ABX + CCI. **C**, **F** **p < 0.01 versus SPF. **H** *p < 0.05 **p < 0.01 versus SPF + CCI. Numbers of animals included in each of the groups: **A–C**, *n* = 6 in SPF, *n* = 10 in SPF + CCI, *n* = 5 in ABX, *n* = 7 in ABX + CCI); **E**, **F**, *n* = 10 in SPF and ABX, *n* = 12 in SPF + CCI and ABX + CCI; **H**, **I**: *n* = 10 in SPF + CCI, *n* = 11 in ABX + CCI

Noxious stimulation-evoked acute pain processing mainly involves neuronal transduction. However, neuropathic and inflammatory pain requires immune activation and neuro-immune interactions [22]. Gut microbiota has been shown to play tremendous roles in regulating the functions of immune systems [2]. We asked whether gut microbiota depletion could alter the development of nerve injury-induced chronic pain. CCI treatment produced long-lasting thermal hyperalgesia and mechanical allodynia in mice. Gut microbiota deletion by pretreatment of ABX for 2 weeks resulted in significant alleviation of CCI-induced thermal hyperalgesia, but not the mechanical allodynia (Fig. 2A–C). We then wondered whether such pre-depletion of gut microbiota was needed to exert its analgesic effect and whether the gut microbiota manipulation could influence the development and persistence of neuropathic pain. We started ABX feeding simultaneously with nerve injury (at day 0 of CCI treatment) and found that the thermal hyperalgesia, but not the mechanical allodynia was also impaired starting within 24 h and lasting to the last test on day 28. The analgesic effect is significant compared with SPF control animals (Fig. 2D–F). We then had ABX feeding starting at day 7 after CCI treatment and found that the established thermal hyperalgesia was reversed and the mechanical allodynia still remained unchanged (Fig. 2G–I). Taken together, these results indicate the influence of gut microbiota on chronic neuropathic pain with several features, i.e., the gut microbiota depletion selectively impacts the pathway of the thermal hyperalgesia, but not that of the mechanical allodynia; the effects of gut microbiota on neuropathic pain could be instant; and that gut microbiota is able to influence both the early development and late maintenance of neuropathic pain.

**Fig. 3:**
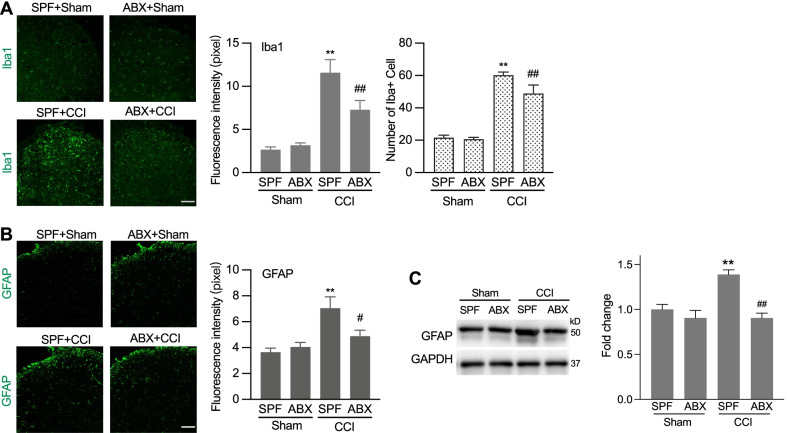
ABX treatment alleviated CCI-induced activation of microglia and astrocyte in the spinal cord of mice. **A** Representative images of the Iba-1 microglia immunostaining (scale bar: 100 µm) and the quantification of the fluorescence intensity (upper) and cell numbers (lower) of the Iba1 immunostained cells. One-way ANOVA with Dunnett multiple comparison test **p < 0.01 versus SPF, ^##^*p* < 0.01 versus SPF + CCI (15 slides from 4 SPF sham mice, 15 slides from 4 ABX sham mice, 12 slides from 4 SPF + CCI mice, and 11 slides from 3 ABX + CCI mice). **B** Representative images of the GFAP astrocyte immunostaining (scale bar: 100 µm) and the quantification of the fluorescence intensity the GFAP immunostaining cells. One-way ANOVA with Dunnett multiple comparison test **p < 0.01 versus SPF, ^#^*p* < 0.05 versus SPF + CCI (48 slides from 4 SPF mice, 51 slides from 4 ABX mice, 28 slides from 4 SPF + CCI mice, and 28 slides from 3 ABX + CCI mice). **C** Representative image of bands and statistical summary of the western blot analysis showing that CCI-induced GFAP increase was reversed by ABX treatment. *n* = 3 in each group. **p < 0.01 versus SPF, ^##^*p* < 0.01 versus SPF + CCI. Tissues were taken from SPF and ABX-treated mice at day 7 after CCI or sham operation (**A**–**C**)

After nerve injury, activation of microglia and astrocyte in the spinal cord is critical for the chronification of pain hypersensitivity [23]. Gut microbiota has been found to shape the maturation of microglia in the spinal cord [19] and the astrocyte functions in the brain [24]. Thus, the microbiota may contribute to the nerve injury-induced thermal hyperalgesia through influencing the glia cell function. We continued to examine the glia cell activation in CCI animals with ABX treatment. Immunofluorescence staining or western blot assay showed that CCI-induced increased activation of microglial cells (Iba1) and astrocytes (GFAP) in the spinal cord was greatly suppressed in animals with ABX treatment. Tissues of the spinal cord was taken on day 7 after CCI treatment (Fig. 3A–C). These results suggest that microbiota may contribute to the nerve injury-induced glial cell activation, which contributes to the development of neuropathic pain.

### Gut microbiota depletion ameliorates mechanical allodynia and inhibits cytokines production in DRG neurons in animals with chemotherapy

**Fig. 4:**
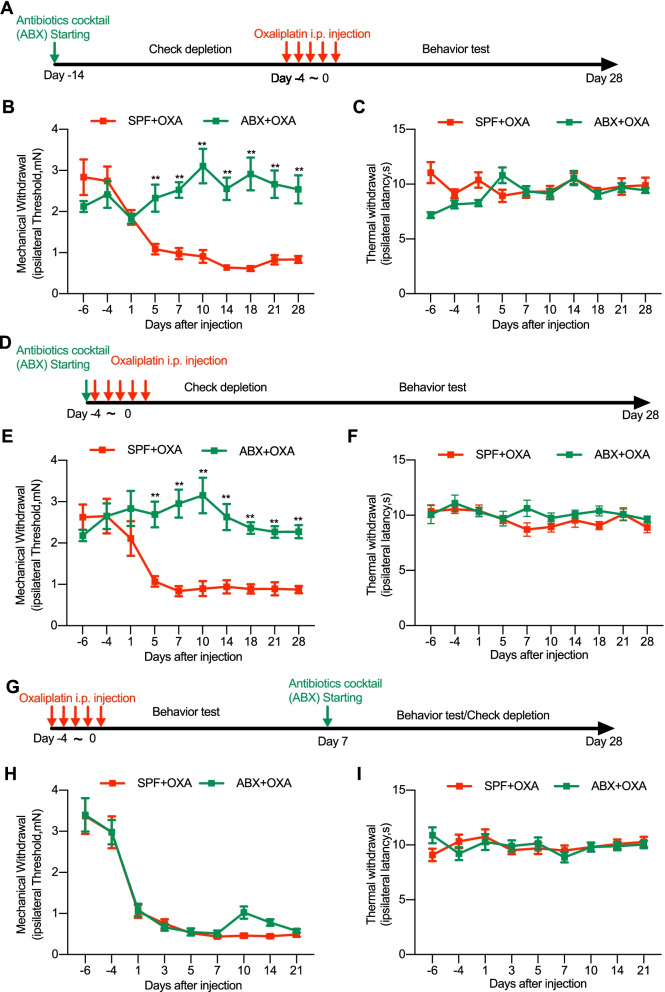
The continuous feeding of ABX alleviates chemotherapy (OXA)-induced mechanical allodynia. OXA treatment itself did not induced significant alteration of thermal sensitivity. **A**–**C** Pre-depletion of gut microbiota, two weeks earlier than OXA treatment. **D**–**F** The continuous feeding of ABX starting simultaneously with OXA treatment. **G**–**I** The continuous feeding of ABX starting two weeks after OXA treatment when the mechanical allodynia had been established. Two-way ANOVA with Sidak multiple comparison test. **p < 0.01, SPF + OXA versus ABX + OXA. Numbers of animals: *n* = 11 in SPF + OXA; *n* = 10 in ABX + OXA)

A recent study shows that gut microbiota depletion by antibiotics or germ-free condition can prevent the development of mechanical pain in chemotherapy-induced europathic pain [13]. It remains unknown how gut microbiota may influence different modalities of pain behaviors and different phases over the time course of the disease in chemotherapy-induced painful hypersensitivity. Therefore, parallel to that we have tested in mice with CCI treatment, we performed similar investigations in mice with chemotherapy-induced neuropathic pain. In those animals with repetitive administration of chemotherapy drug oxaliplatin (OXA) (i.p., daily for 5 consecutive days), ABX feeding was started prior to (from 2 weeks before), simultaneously at the time point of, or 7 days after OXA treatment. Unlike CCI, OXA treatment induced long-lasting behaviorally expressed mechanical allodynia without thermal hypersensitivity (Fig. 4A–I). Pretreatment of ABX feeding starting 2 weeks prior to OXA completely inhibited the development of mechanical allodynia (Fig. 4B), which is consistent to the previous report [50]. What’s more, our results further showed that ABX feeding starting simultaneously with OXA treatment also completely prevented the development of mechanical allodynia (Fig. 4E). However, ABX feeding after OXA-induced mechanical allodynia had established did not produce significant analgesic effect on the mechanical allodynia, but a temporary and slight inhibitory effect 3 days after the ABX treatment (Fig. 4H). ABX treatment did not show any additional effect on the thermal sensitivity (Fig. 4C, F and I). These results indicate that gut microbiota depletion can prevent development and persistence of OXA-induced mechanical allodynia, suggesting that microbiota may contribute to the chemotherapy-induced chronic pain.

**Fig. 5:**
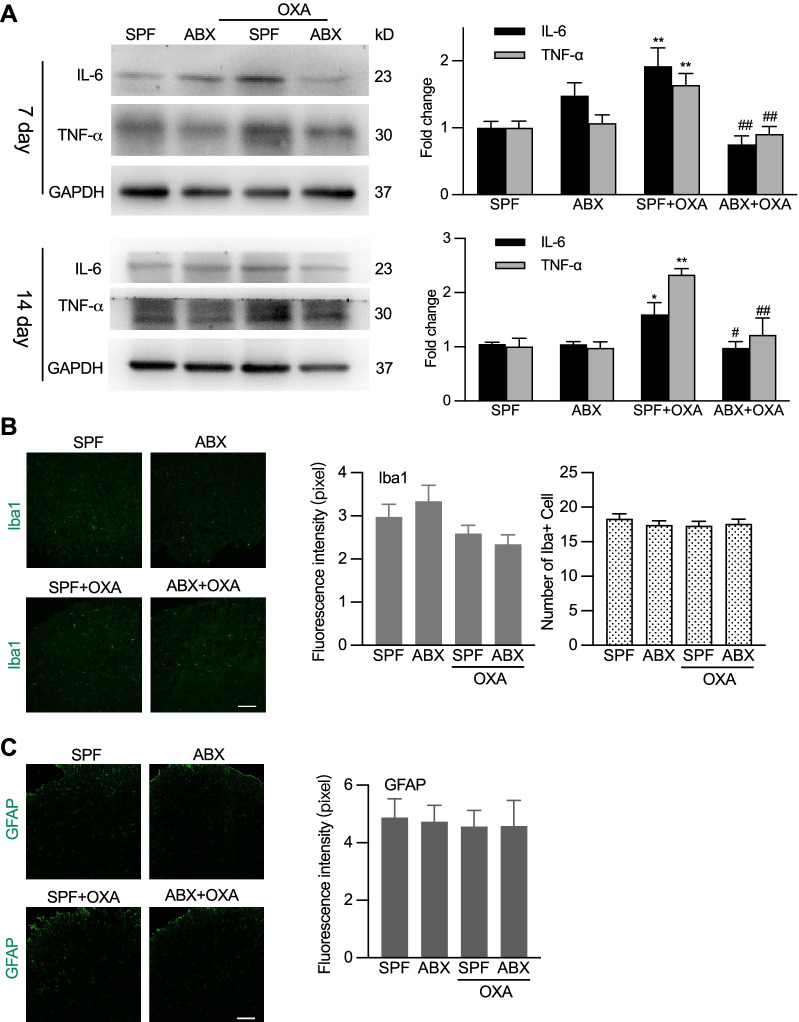
Differential effects of ABX treatment on chemotherapy-induced cytokine production in DRG and glia cell activation in the spinal cord. **A** Western blotting analysis showing that ABX treatment suppressed the expression of IL-6 and TNF-α in DRG in day 7 and 14 after OXA treatment. Left: representative Western blot bands. Right: statistical summary. One-way ANOVA with Tukey multiple comparison test *p < 0.05, **p < 0.01 SPF + OXA versus SPF; ^#^*p* < 0.05, ^##^*p* < 0.01 ABX + OXA versus SPF + OXA (on day 7: IL-6, *n* = 6 in each group; TNF-α, *n* = 7 in ABX, *n* = 6 in other groups, and on day 14: *n* = 3 in each group). **B**, **C** Both OXA and ABX treatment did not alter the activation of microglial cells and astrocytes in the spinal cord. Left: representative images of immunostaining of Iba1 and GFAP. Scale bar: 100 µm. Right: quantification of the fluorescence intensity and cell numbers of Iba1 immunostaining. Tissues were taken 7 days after OXA treatment (**B** and **C**). In **B**, 15 slides from 4 mice in SPF, 16 slides from 4 mice in ABX, 16 slides from 4 mice in SPF + OXA, 17 slides from 4 mice in ABX + OXA. In **C**, 6 slides from 4 mice in each group

Different from nerve injury, studies have shown that OXA chemotherapy does not cause glia cell activation and glial cell activation does not contribute to OXA chemotherapy-induced neuropathic pain [25]. We here confirmed that both microglial cells and astrocytes in the spinal cord were not activated following OXA treatment with or without ABX treatment (Fig. 5A, B). These findings may help to exclude the possible roles of glia cell activation in chemotherapy-induced neuropathic pain and microbiota-depletion-caused alterations of sensory sensitivity. It was previously reported that gut microbiota facilitated recruitment of cytokines producing macrophage into DRG after chemotherapy [13]. We thus examined some macrophage producing cytokines in DRG by western blot. The results showed OXA treatment resulted in greatly increased expression of IL-6 and TNF-α, while ABX treatment caused long-term inhibition of OXA-induced high expression of cytokines, both at day 7 and 14 after OXA administration (Fig. 5C). These findings demonstrate that in chemotherapy-induced neuropathic pain the gut microbiota facilitates the cytokine production and immune functions in DRG and that gut microbiota may influence chemotherapy- and nerve injury-induced neuropathic pain in distinct mechanisms.

### Gut microbiota depletion prevents development of STZ-induced diabetes and mechanical allodynia and inhibits glia cell activation in the spinal cord

**Fig. 6:**
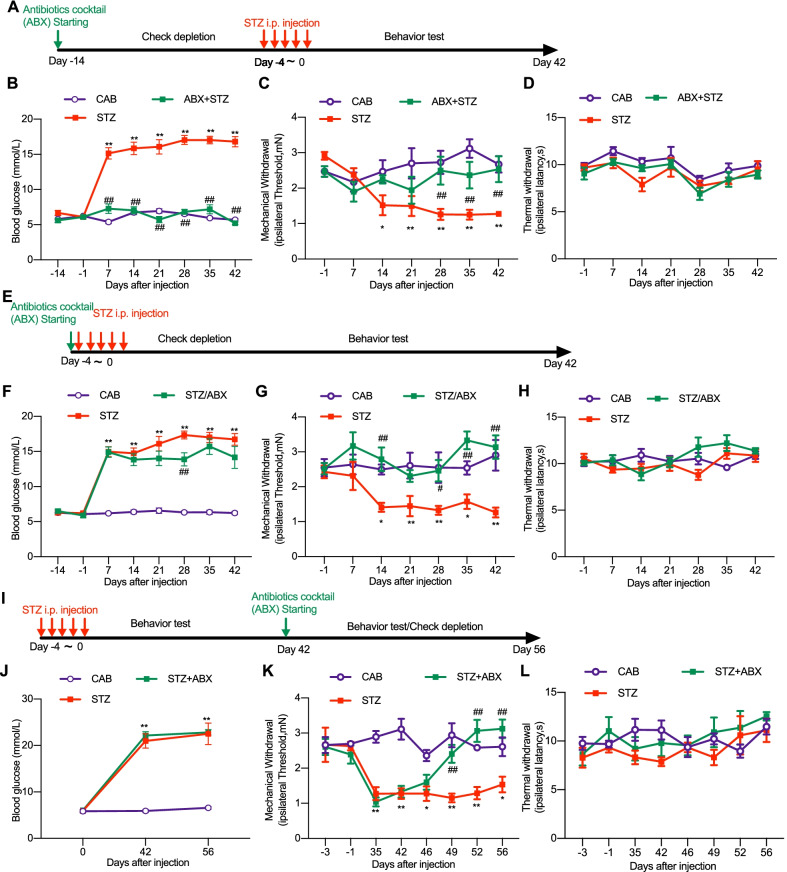
Differential effects of continuous feeding of ABX on STZ-induced diabetic high blood glucose and mechanical allodynia. STZ treatment itself did not induced significant alteration of thermal sensitivity. **A**–**D** Pre-depletion of gut microbiota, with continuous feeding of ABX starting two weeks earlier than STZ treatment (**A**), prevented STZ-induced high blood glucose (**B**) and mechanical allodynia (**C**), without affecting the thermal sensitivity (**D**). **E**–**H** The continuous feeding of ABX starting simultaneously with STZ treatment (**E**) reduced STZ-induced mechanical allodynia (**G**) without significantly affecting the high blood glucose (**F**) and the thermal sensitivity (**H**). **I**–**L** The continuous feeding of ABX starting 42 days after STZ treatment (**I**) when the high blood glucose and mechanical allodynia had been established suppressed the mechanical allodynia (**K**) without affecting the high blood glucose (**J**) and the thermal sensitivity (**L**). Two-way ANOVA with Dunnett multiple comparison test *p < 0.05, **p < 0.01, STZ versus citric acid buffer (CAB) control. ^#^*p* < 0.05, ^##^*p* < 0.01, STZ + ABX versus STZ alone. Numbers of animals: in **B**–**D**, *n* = 6 in CAB; *n* = 12 in STZ at day 21 and 28, *n* = 11 at day 14, *n* = 13 at other data points; in ABX + STZ, *n* = 16 (**B**) and *n* = 8 (**C** and **D**). In F–H, *n* = 6 in CAB; *n* = 13 in STZ; in STZ + ABX, *n* = 7 at day 42, *n* = 9 at day − 7. In J-L, *n* = 8 in each of the groups

Diabetic neuropathic pain (DNP) is another common form of neuropathic pain. We investigated whether gut microbiota could be involved in development of DNP. STZ causes selective destruction of pancreatic islet β-cell, followed by insulin deficiency and hyperglycemia, which are characteristic of human type 1 diabetes mellitus[16]. Repetitive administration of STZ (i.p., 40 mg/kg, one dose per day for five consecutive days) (Fig. 6A) produced significant and persistent increase of blood glucose level (Fig. 6B) accompanied with mechanical allodynia, but not thermal hypersensitivity (Fig. 6C, D). Surprisingly, pretreatment of ABX feeding for two weeks completely prevented STZ-induced increase of blood glucose (Fig. 6B) and no mechanical allodynia or thermal hypersensitivity was shown in these mice due to no diabetes onset. (Fig. 6C, D). These results show that pre-depletion of gut microbiota can completely prevent the development of high blood glucose, suggesting that gut microbiota may play a protective role in the STZ-induced destruction of pancreatic islet β-cell.

**Fig. 7:**
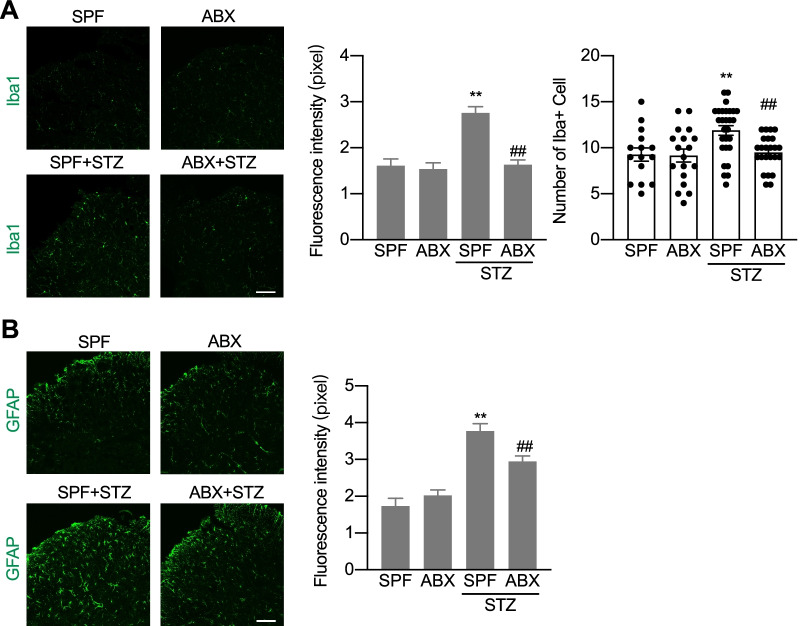
ABX treatment reduces STZ-induced activation of microglia and astrocyte in the spinal cord of mice. **A** Representative images of the Iba-1 microglia immunostaining (scale bar: 100 µm) and the quantification of the fluorescence intensity (upper) and cell numbers (lower) of the Iba1 immunostaining cells. One-way ANOVA with Dunnett multiple comparison test **p < 0.01 versus SPF, ^##^*p* < 0.01 versus SPF + STZ (15 slides from 4 SPF and ABX mice, 28 slides from 4 mice with SPF + STZ, and 24 slides from 4 mice with ABX + STZ). **B** Representative images of the GFAP astrocyte immunostaining (scale bar: 100 µm) and the quantification of the fluorescence intensity of the GFAP immunostaining cells. One-way ANOVA with Dunnett multiple comparison test **p < 0.01 versus SPF, ^#^*p* < 0.05 versus SPF + STZ (21 slides from 4 SPF mice, 24 slides from 4 ABX mice, 22 slides from 4 SPF + STZ mice, and 23 slides from 4 ABX + STZ mice). Tissues were taken from SPF and ABX-treated mice at day 42 after STZ or sham operation (**A** and **B**)

We continued to repeat the experimental protocols in STZ model as those used in CCI and OXA models. ABX feeding simultaneously with STZ treatment (Fig. 6E) slightly decreased STZ-induced high blood glucose (Fig. 6F) and permanently completely diminished the mechanical allodynia (Fig. 6G) without affecting the thermal sensitivity (Fig. 6H). ABX feeding starting 42 days after STZ treatment (Fig. 6I) did not affect the established high blood glucose (Fig. 6J), but did successfully suppressed the established mechanical allodynia (Fig. 6K) and had no effect on the thermal sensitivity (Fig. 6L). Glia cell activation has been reported to play roles in diabetic neuropathic pain [26]. We continued to examine the glia cell activation in the spinal cord in STZ-treated animals with high blood glucose and mechanical allodynia with or without ABX treatment. The immunostaining showed that expression of Iba1 and GFAP in the spinal cord was significantly increased 2 weeks after STZ treatment. Simultaneous feeding of ABX with STZ treatment partially but significantly decreased STZ-induced increased expression of Iba1 and GFAP (Fig. 7A–D). Taken together, these results demonstrate that the gut microbiota is required for the production, but not the persistence of STZ-induced diabetes, which was characterized as the high blood glucose. Meanwhile, the gut microbiota is required for both induction and maintenance of STZ-induced mechanical allodynia.

### Fecal bacteria transplantation partially reverses the gut microbiota colonization and fully rescues gut microbiota depletion-induced behaviorally expressed pain in CCI, OXA, and STZ models

**Fig. 8:**
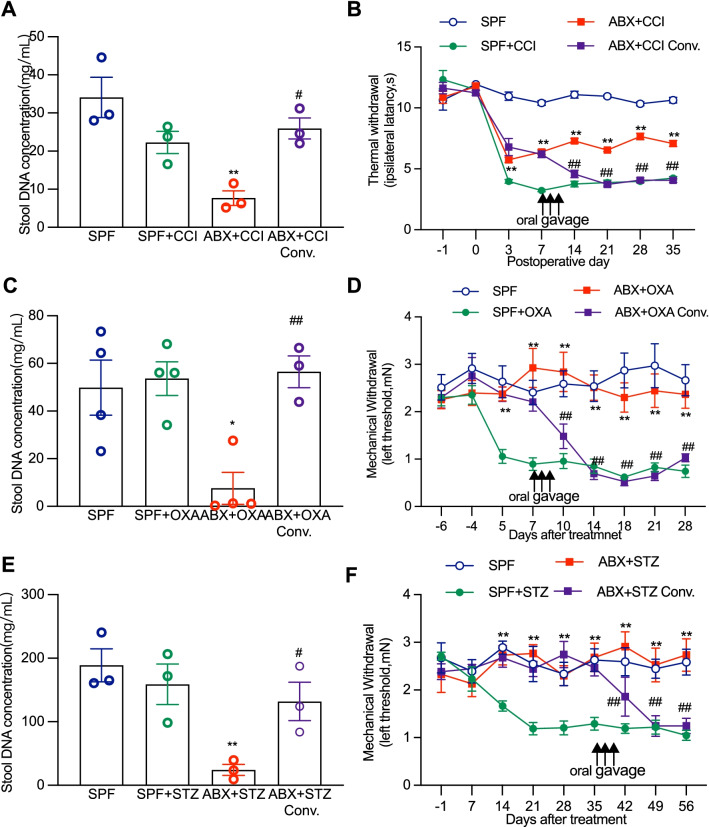
Oral gavage of fecal microbiota from SPF mice restores ABX-induced gut microbiota depletion and rescues neuropathic pain. Fecal bacteria conventionalization was used to restore the depleted gut microbiota and the successful restoration of fecal microbiota was verified by detecting fecal microbiota DNA concentration. **A**–**F** ABX-induced gut microbiota depletion and analgesic effects were restored and reversed following fecal bacteria conventionalization in the animals that received treatment of CCI (**A**, **B**), OXA (**C**, **D**) and STZ (**E**, **F**). Arrows show the timing of fecal bacteria conventionalization and ABX withdrawal. Statistical analysis, one-way ANOVA (**A**, **C**, **E**) or two-way ANOVA with Dunnett multiple comparison (**B**, **D**, **F**), **A** **p < 0.01 versus SPF, ^#^*p* < 0.05 versus ABX + CCI (*n* = 3 in each group). **B** **p < 0.01 versus SPF + CCI, ^##^*p* < 0.01 versus ABX + CCI (*n* = 9 at day 7 in ABX + CCI conv, *n* = 12 at day -1/0/3, *n* = 7 at other data points, *n* = 10 in other group). **C** *p < 0.05 versus SPF, ^##^*p* < 0.01 versus ABX + OXA (*n* = 4 in SPF, *n* = 4 in SPF + OXA, *n* = 4 in ABX + OXA, *n* = 3 in ABX + OXA conv.). **D** **p < 0.01 versus SPF + OXA, ^##^*p* < 0.01 versus ABX + OXA (*n* = 10 in SPF and ABX, *n* = 11 in ABX + OXA, *n* = 6 in ABX + OXA conv at day 10, *n* = 5 at day 14/18/21/28, *n* = 10 at other data points). **E** **p < 0.01 versus SPF, ^#^*p* < 0.05 versus ABX + STZ (*n* = 3 in each group). **F** **p < 0.01 versus SPF + STZ, ^##^*p* < 0.01 versus ABX + STZ (*n* = 6 in CAB, *n* = 7 in ABX + STZ, *n* = 8 in others group)

Given that gut microbiota depletion can greatly reduce or completely diminish the painful thermal hyperalgesia or mechanical allodynia caused by nerve injury, chemotherapy, and diabetes, we asked whether restoration of gut microbiota in ABX-treated animals could reverse the painful behaviors in CCI, OXA and STZ animals. ABX feeding was started from 14 days prior to CCI, OXA, or STZ treatment and then was withdrawn by replacing ABX with normal water at day 7 after CCI, day 14 after OXA, and day 35 after STZ treatment followed immediately by oral gavage with fresh fecal bacteria solution taken from SPF mice (a dose per day for 3 continuous days). Feces of the mice were collected at the end time point of the behavioral tests and the fecal bacteria DNA concentration was measured. The results showed that fecal bacteria transplantation did recover ABX treatment-induced depletion of gut microbiota colonization and alterations of sensory sensitization in animals with CCI, OXA or STZ treatment (Fig. 8A–F). In CCI mice, ABX treatment depleted gut microbiota and produced limited but significant inhibitory effect on the thermal hyperalgesia. Such gut microbiota depletion and thermal hypersensitivity reduction were completely reversed following fresh fecal bacteria transplantation (Fig. 8A, B). In mice with OXA or STZ treatment, ABX treatment depleted gut microbiota and prevented or completely suppressed the mechanical allodynia. Again, the treatment with fresh fecal bacteria transplantation quickly and completely restored the gut microbiota and recovered the mechanical hypersensitivity (Fig. 8C–F).

**Fig. 9:**
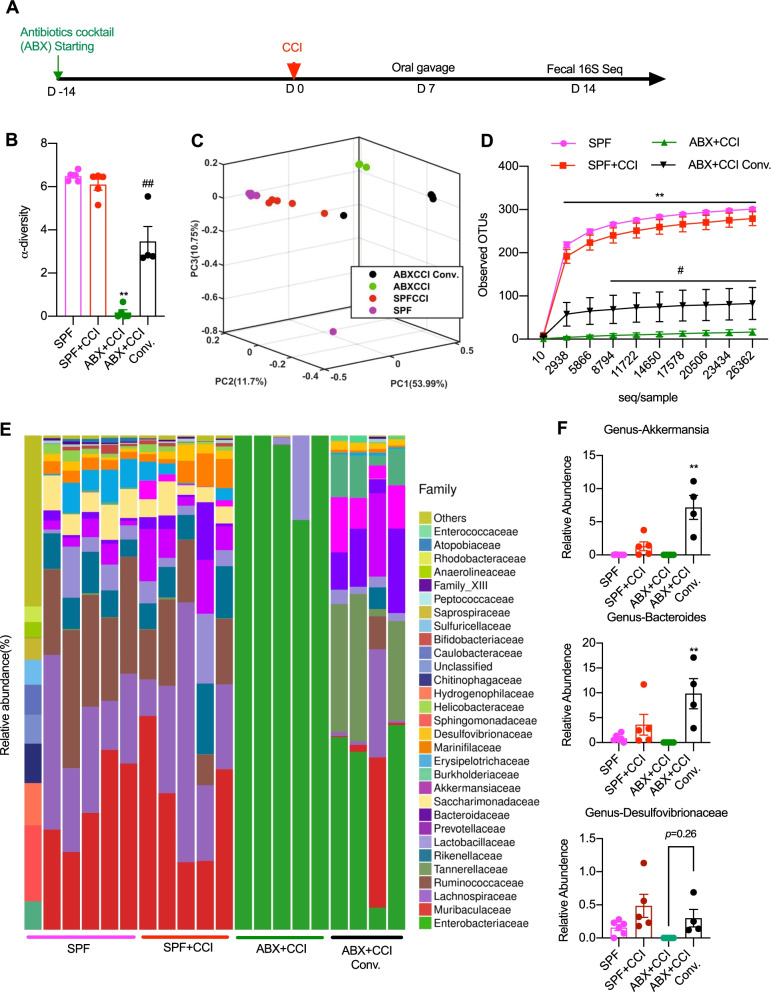
16S rRNA sequencing profiles of the gut microbiota in SPF control mice and those with CCI and ABX treatment as well as restoration of gut microbiota. **A** Timeline of experimental design. **B** α-diversity (Shannon) of the composition of microbiota. One-way ANOVA with Dunnett multiple comparison, **p < 0.01 versus SPF; ^##^*p* < 0.01 versus ABX + CCI. **C** PCA analysis showing the major differences in the composition of microbiota. **D** Observed operational taxonomic units (OUTs) of microbiota. Two-way ANOVA with Tukey multiple comparison **p < 0.01 versus SPF + CCI, #*p* < 0.05 versus ABX + CCI. **E** Stacked bar-chart showing gut microbiota composition at family level. **F** Specific bacteria genus abundance that corelated with the restoration of pain hypersensitivity. Oral fecal microbiota gavage increased the relative abundance of Akkermansia genus (upper), Desulfovibrionaceae genus (middle), and Bacteroides genus (bottom). One-way ANOVA with Tukey multiple comparison ^##^*p* < 0.01 versus ABX + CCI. Number of animals: *n* = 6 in SPF, *n* = 5 in SPF + CCI, *n* = 5 in ABX + CCI, and *n* = 4 in ABX + CCI Conv

To compare the detailed composition of gut microbiota in fecal bacteria transplanted mice with that from SPF mice, we performed 16S rRNA sequencing analysis in mice with CCI treatment. The treatment protocol is shown in Fig. 9A. The sequencing results showed that fecal bacteria transplantation induced a significant restoration of the microbiota from ABX-treated mice (Fig. 9B–E). The restoration of the microbiota community was indicated by the increase of the α-diversity of the composition of the microbiota (Fig. 9B), separation of transplanted mice feces from ABX-treated mice in PCA analysis (Fig. 9C), and increased OTU counting (Fig. 9D). Nevertheless, the composition of the microbiota of the mice that received fecal bacteria transplantation was still significantly different from that from SPF mice (Fig. 9F). The transplanted mice microbiota showed lower α-diversity, separation from SPF mice in the PCA analysis and lower OTU counting than that in SPF mice (Fig. 9B–D). In order to link the possible relationship of some specific bacteria families with nerve injury-induced painful behaviors, we further examined the abundance of Akkermansia, Bacteroides, and Desulfovibrionaceae phylus in the feces of mice with nerve injury, CCI treatment. The results showed that the abundance of these three bacteria was restored or even over expressed in fecal bacteria transplanted mice and positively related to the extent of painful behaviors (Fig. 9F), indicating their possible specific roles in pain regulation.

Taken together, our results demonstrate that effects of gut microbiota on neuropathic pain behaviors are reversible simply by microbiota transplantation and that the restoration of behaviorally expressed painful syndromes does not require fully restoration of gut microbiota, suggesting that certain bacteria or sub-community of the microbiota may be specifically responsible for the pain behavior regulation. Akkermansia, Bacteroides, and Desulfovibrionaceae phylus may belong to the important gut microbiota, which are important to the development of painful manifestation in the nerve injured-mice.

## Discussion

Our study reveals critical roles of gut microbiota in the development and maintenance of neuropathic pain in mice with peripheral nerve injury, chemotherapy, and diabetic neuropathy. Production and persistence of neuropathic pain induced by CCI, OXA, and STZ, respectively, can be prevented or greatly suppressed by gut microbiota depletion and rescued following gut microbiota restoration by transplanting fresh fecal bacteria from SPF animals. Continuous feeding of antibiotics cocktail caused major depletion of the gut microbiota. Gut microbiota depletion prevents or completely suppresses CCI-, OXA-, and STZ-induced thermal hyperalgesia or mechanical allodynia as well as inhibiting CCI or STZ treatment-induced glial cell activation in the spinal cord and OXA-induced cytokine production in DRG. Gut microbiota depletion can also prevent STZ from inducing the increase of blood glucose, but had no effects on the established high blood glucose. More importantly, gut microbiota restoration by transplanting fresh fecal bacteria from SPF animals can rescue the neuropathic pain following the nerve injury or diseases, of which, Akkermansia, Bacteroides, and Desulfovibrionaceae phylus may play a key role. These findings demonstrate distinct roles of gut microbiota in the pathogenesis of chronic pain following nerve injury, chemotherapy and diabetic neuropathy and support the clinical significance of fecal bacteria transplantation. The possibility of altering neuropathic painful sensitivity by fecal transplantation, probiotics or prebiotics may extend the new avenue of pain management by manipulating the gut microbiota.

Gut microbiota, which represents the massive commensal microbes colonizing the gastrointestinal (GI) tract, have been demonstrated to play important roles in many physiological and pathological processes of our bodies and regulate mucosal immune functions of the gut, GI tract motility, and digestive processes. Recent studies have also shown that gut microbiota participates in the development of inflammation pain [12] and chemotherapy-induced neuropathic pain [13]. Our study here systematically investigated the roles of gut microbiota in different forms of chronic pain conditions and demonstrates the involvement of gut microbiota in the pathogenesis of chronic pain following nerve injury, chemotherapy, and diabetes, in timing and modality specific manners. The established gut microbiota depletion can prevent the production of neuropathic pain and greatly suppress the established neuropathic pain. Late depletion of gut microbiota can suppress CCI- and STZ-, but not OXA-induced established pain. Consistently, the neurochemical signs of CCI-, OXA- and STZ-induced neuropathic pain the glial cell activation in the spinal cord and cytokine production in DRG can be inhibited by the gut microbiota depletion. These findings provide new and important evidence supporting the critical roles in the pathogenesis of chronic pain after different forms of injury or diseases and extend our understanding of how gut microbiota is able to play important roles in diverse functions of our body.

Interestingly, we have also found that microbiota depletion can completely prevent the development of STZ-induced diabetes manifested as the high blood glucose. This unexpected finding provides a promising evidence that gut microbiota may play critical roles both in the development of STZ-induced diabetes itself and diabetic neuropathic pain symptoms. Further studies are urgently needed for understanding its roles in the pathogenesis of diabetes and in diabetic pain.

Another important finding in this study is that partial restoration of gut microbiota can fully rescue the neuropathic pain, which was previously prevented or suppressed by gut microbiota depletion. Of which, Akkermansia, Bacteroides, and Desulfovibrionaceae phylus may play important roles in the development of painful manifestation in the nerve injured-mice. In order to extend the avenue of pain management by manipulating gut microbiota, we need to find the precise links between gut bacteria and neuronal pathways. The interactions of the nervous system with some other specific bacteria of the gut microbiota has previously been identified. By circulation of microbial metabolites or altering neuronal transduction by vagus nerve, the gut microbiota is able to interact with systems that are distal to the gut, including DRG, the spinal cord and the brain. It has been found that LPS from Gram-negative bacteria in the gut is released into the blood and circulates into DGR and the spinal cord [13]. The activation of Toll-like receptors by LPS is very important in the neuron–immune interactions and pain processing in the nervous system [27]. Short chain fatty acids produced by bacteria were found to be critical to the maturation of the microglia [19]. Cytokines produced in the gut, which are highly impacted by gut microbiota, are able to regulate the function of astrocytes in the central nervous system [24]. Bacteroides fragilis of gut microbiota produces sphingolipids [28] and polysaccharides [29] into the circulation, which plays important roles in neuronal myelination [30], neuroinflammation [31], neuronal excitability[29], and chronic pain [32]. Besides microbial metabolites, the gut microbiota is also capable of regulating the levels of some neurotransmitters or neuromodulators, such as GABA [33] and serotonin (5-HT) [8], to impact on the neuronal functions. These findings extend our understanding of how gut microbiota could interact with the nervous system. Our study also provides a possibility of how specific bacteria of gut microbiota could interact with the pain processing in the spinal cord and DRG, but further identification of these interactions is needed.

Several lines of studies have demonstrated that gut microbiota is essentially an organic part of our body. Gut microbiota plays important roles in digestive processes, immune functions, as well as neuronal functions and in the pathogenesis of certain diseases including cancer therapies [34], diabetes [35], and neurological diseases like Alzheimer’s disease [11] and autism spectrum disorders [7]. The gut is a very complicated interface where the nervous system, immune system and the gut commensal microbes all reside in and interact with each other. The gut microbiota is not in proximity to the transduction pathway of the somatic pain sensation, especially of the sensation of the skin or limbs. How the gut microbiota is able to distally regulate the somatic pain sensations remains unknown. Studies have shown that the gut is heavily innervated by primary sensory nerves (both DRG and nodose nerve), sympathetic and parasympathetic nerves. Besides these external nerves, the gut also hosts a relatively independent internal enteric nervous system. As a direct interface between the internal body and external environment, the gut is very active in immune activities, featuring different kinds of innate an adaptive immune cell. All these complexities of different systems add up and increases the possibility of interesting interactions among them. In summary, our study reveals distinct roles of gut microbiota in different forms of somatic chronic pain. Further studies are needed to explore the impact of gut microbiota on glial functions and neuron–immune interactions in pain processing.

## Conclusions

Gut microbiota depletion by antibiotics suppresses thermal hyperalgesia or mechanical allodynia in nerve injury, chemotherapy- or diabetic neuropathy-induced chronic pain conditions. Reduced painful behaviors may be due to decreased glia activation or altered cytokine production in the spinal cord or DRG. Fecal microbiota transplantation rescues gut microbiota depletion-induced pain behavior changes, of which certain bacteria shows correlation with the pain behaviors. In brief, this study demonstrates distinct roles of gut microbiota in the pathogenesis of chronic painful conditions with nerve injury, chemotherapy and diabetic neuropathy and supports the clinical significance of fecal bacteria transplantation.

## Data Availability

All data related are included in the manuscript and will be available from the corresponding author on reasonable request.

## Abbreviations

CCI: Chronic constriction injury
OXA: Oxaliplatin
STZ: Streptozocin
ABX: Antibiotics
DRG: Dorsal root ganglion
GI: Gastrointestinal
LPS: Lipopolysaccharides
SPF: Specific pathogen free
PBS: Phosphate buffered saline
i.p.: Intraperitoneally
CFU: Colony forming unit
OTU: Operational taxonomic unit
GAPDH: Glyceraldehyde 3-phosphate dehydrogenase
GFAP: Glial fibrillary acidic protein
IBA1: Ionized calcium binding adaptor molecule 1
RIPA: Radioimmunoprecipitation assay buffer
ELISA: Enzyme linked immunosorbent assay
TSA: Tryptic soy agar
PCA: Principal component analysis
EC: Enterochromaffin cell
IL-6: Interleukin 6
TNF-α: Tumor necrosis factor alpha
DNP: Diabetic neuropathic pain
